# International Price Comparisons of Anticancer Drugs: A Scheme for Improving Patient Accessibility

**DOI:** 10.3390/ijerph18020670

**Published:** 2021-01-14

**Authors:** Jae Ho Jung, Dae Jung Kim, Kangho Suh, Jaeeun You, Je Ho Lee, Kyung In Joung, Dong Churl Suh

**Affiliations:** 1 College of Pharmacy, Chung-Ang University, Seoul 06974, Korea; Jaeho.jung@novartis.com (J.H.J.); daejung@daiichisankyo.co.kr (D.J.K.); Wodms5883@gmail.com (J.Y.); ouykk@hanmail.net (J.H.L.); 2 Novartis Korea Ltd., Seoul 07326, Korea; 3 Department of Pharmacy & Therapeutics, University of Pittsburgh School of Pharmacy, Pittsburgh, PA 15217, USA; kas551@pitt.edu; 4 Graduate School of Public Health, Seoul National University, Seoul 08826, Korea

**Keywords:** price comparison, anticancer drugs, international price, price index, patient access

## Abstract

Background: The demand for implementing a new listing scheme to expedite patient access to novel oncology drugs has increased in South Korea. This study was conducted to compare the prices of anticancer drugs between eight countries and to explore the feasibility of a ‘pre-listing and post-evaluation’ scheme to expedite patient access to oncology drugs. Methods: This study included 34 anticancer drugs, which were reimbursed between 1 January 2007 and 31 December 2017. The unit price and sales volume of the study drugs were collected from eight countries and IQVIA data, respectively. The prices were adjusted to estimate the ex-factory prices using the discount/rebate rate suggested by the Health Insurance Review Agency (HIRA). The four price indices of Laspeyres, Paasche, Fisher, and the unweighted index were calculated using the price in each country, the average price, and lowest price among the study countries. Each currency was converted using the currency exchange rate and purchasing power parity (PPP). The budget impact of implementing the proposed pre-listing and post-evaluation scheme on payers was calculated. Results: Based on the currency exchange rate, anticancer drug prices were higher in other countries (index range: 1.05–2.78) compared to Korea. The prices in Korea were similar to countries with the lowest prices. When the PPP was applied, prices were higher in the US, Germany, Italy, and Japan than in Korea (range: 1.10–2.13); however, the prices were lower in the UK, France, and Switzerland than in Korea (range: 0.72–0.99). The financial burden of implementing the pre-listing and post-evaluation scheme was calculated at 0.83% of the total anticancer drug sales value in Korea from 2013–2017. Conclusions: The prices of anticancer drugs in Korea were similar to the lowest prices among the seven other study countries. A pre-listing and post-evaluation scheme should be considered to improve patient access to novel anticancer drugs by reducing the reimbursement review time and uncertainties.

## 1. Introduction

Cancer is the second leading cause of death worldwide, accounting for 16.6% of all deaths (9.6 million deaths) in 2018 [1]. By 2040, the number of new annual cancer cases is expected to increase to 29.5 million, and the number of cancer-related deaths is estimated at 16.4 million [2]. As such, the economic burden of cancer is significant and increasing globally. In South Korea, the cancer prevalence per 100,000 population has increased by 70% from 1450 to 2472 between 2010 and 2017 [3]. Cancer-related mortality rates have decreased over the last several decades due to significant advances in the development of novel anticancer drugs [4]. The number of anticancer drugs approved by the Food and Drug Administration (FDA) in the United States (USA) has increased substantially in the past 10 years, from four in 2010 to 12 in 2019; however, the number of approved anticancer drugs in Korea has only increased slightly from eight to 10 in the same period [5,6].

Despite recent innovations in oncology treatments, the high cost of new anticancer drugs and uncertain clinical outcomes present a dilemma for decision makers and a potential barrier to access. These challenges, complemented by patient demands for fast access to new therapies, have prompted countries to develop new pricing and reimbursement policies [7]. Managed entry agreements, also called risk-sharing agreements or patient access schemes, are general policy mechanisms that allow public payers to balance accessibility and affordability for costly new drugs and have become more popular in several countries [8]. However, the implementation of these agreements is inconsistent across countries due to differences in health care systems and national economies [7,9,10]. The Korean government implemented a risk-sharing agreement with a diverse typology in 2014 [11]; however, the number of novel oncology drugs that meet the strict application criteria is limited.

There is a wide temporal gap between anticancer drug approval and reimbursement. The Korean government spent an average of 601 days to make a reimbursement decision for new anticancer drugs between 2009 and 2014 [12]. Although there are large variations in the time to reimbursement compared with that in other countries, the median period for oncology drugs is still significantly longer in Korea than in other countries: 89 days in Canada, 212 days in France, 320 days in Spain, 321 days in the United Kingdom (UK), and 422 days in Italy [13,14]. Many cancers have a higher chance of being cured if diagnosed and treated early, so creating policies to expedite patient access to advanced anticancer drugs is critical for saving lives and reducing the overall societal economic burden.

Drug pricing determination is a complex process involving policies, structures, and national philosophies, and it varies depending on the drug, which is challenging when comparing drug prices between countries. An objective assessment of drug prices is necessary to balance patient access and affordability while ensuring a sustainable insurance system. In addition to the delayed listing of anticancer drugs in Korea being a major health issue, studies focused on drug prices in Korea are limited; however, these studies have been widely conducted in European countries and the USA [15,16,17]. Furthermore, the results from previous studies that have compared drug prices between Korea and other countries are inconsistent. The Korean government and stakeholders are currently discussing several ways of expediting patient access to new drugs through reducing the time spent on the reimbursement decision making process and making the drugs available at a reasonable price for patients.

To the best of our knowledge, no previous study has suggested a new reimbursement scheme that can enhance patient access to new anticancer drugs through early listing in the national drug formulary while examining its potential financial impact on payers. The objectives of this study were to compare the prices of anticancer drugs among 8 countries including Korea based on price indices, which were calculated using currency exchange rates and purchasing power parities (PPPs), to explore the feasibility of the proposed pre-listing and post-evaluation scheme, and to calculate the budget impact on payers if this new reimbursement scheme is implemented in South Korea.

## 2. Materials and Methods

### 2.1. Selection of Anticancer Drugs and Countries

**Figure 1:**
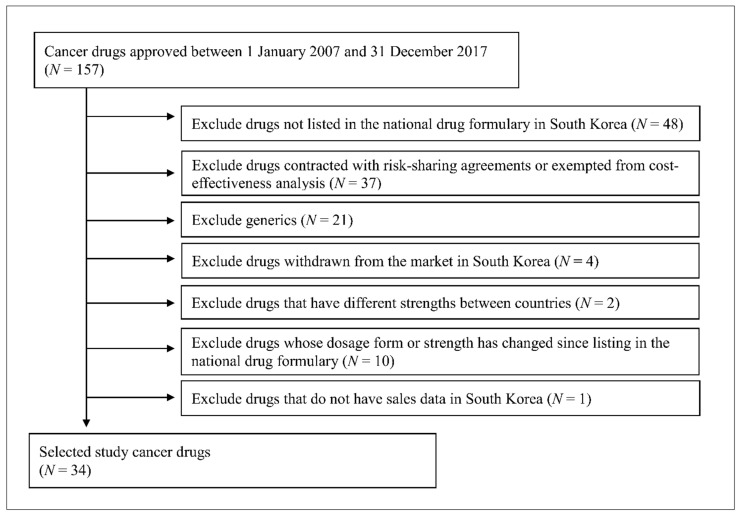
Flow diagram of study drug selection.

This study included 34 originator anticancer drugs, which were classified as 421 (malignant cancer drug) or 429 (other cancer drugs) according to the drug classification codes of the Korean Ministry of Food and Drug Safety and were approved between 1 January 2007 and 31 December 2017 (Table 1). The present study excluded drugs based on the following criteria: if they were not listed in the national drug formulary handled by the Health Insurance Review & Assessment Service (HIRA) because these drug prices were not available, if they were generic drugs because their listing prices were determined based on the originator price, if they were contracted with risk-sharing agreements or exempted from cost-effectiveness analysis because the actual contracted price is confidential, if they were withdrawn from South Korea’s market, and additional criteria. The flow diagram of study drug selection is shown in Figure 1.

Table 1 provides background information on the selected study drugs. The prices and sales volumes of the selected drugs were collected for seven countries; USA, UK, Germany, France, Italy, Switzerland, and Japan; referred to as ‘A7’. The HIRA has used the prices in A7 countries for external reference pricing [18].

The elapsed time in months from the approval date by the Korean Ministry of Food and Drug Safety until the date of listing in the national drug formulary for reimbursement by the Ministry of Health and Welfare was calculated (Table 1).

### 2.2. Data Sources

Drug price data for the A7 countries and South Korea were collected from their national databases in November 2018: South Korea (http://www.hira.or.kr/), USA (https://redbook.solutions.aap.org/redbook.aspx), UK (http://www.mims.co.uk), Germany (http://online.rote-liste.de/), France (http://www.evidal.fr), Italy (http://www.codifa.it), Switzerland (http://www.compendium.ch/search/de), and Japan (https://www.e-pharma.jp/search/). Drug prices for Korea were the list prices at the time of listing in the national drug formulary. They were the official list prices without any consideration of discounts or rebates. Table 2 shows the list prices of these drugs in USA dollars. The list price of each drug in each country was adjusted based on the algorithm of foreign-adjusted average prices suggested by the HIRA. The unit price was defined as the price of one tablet or one vial (or bottle). The strength of each product was based on the strength approved for use in Korea.

In order to determine the ex-factory prices of anticancer drugs, the collected list prices from A7 countries were adjusted to account for a value-added tax (10%) and a sales mark-up suggested by the HIRA [18,19]. The ex-factory prices were assumed to be 65% of the web-based list prices in the USA, the UK, France, Italy, and Switzerland and 82% of those in Japan. The prices in Germany were calculated by referring to the legal profit limit; the adjusted prices were calculated from the list prices without the value-added tax (19%), pharmacy profit (3% and 8.1 euro), and wholesaler profit depending on the price [20]. The sales mark-up consisted of a legal wholesale distribution profit of 1.0869 set by the Korean government [21].

The drug prices in the listing year were adjusted to equivalent prices in 2020 using each country’s consumer price index or the prescription drug price index if that price index was available (for the USA only). The currencies of the A7 countries were converted to US dollars using the average currency exchange rates at the time of listing the drugs in Korea and the PPPs provided by the Organisation for Economic Co-operation and Development (OECD) [22]. The currency rates equivalent to 1 US dollar were as follows: Korea (1164.45), the UK (0.765), Germany (0.900), France (0.852), Italy (0.900), Switzerland (0.970), and Japan (109.26). Currency conversion using PPPs is an alternative method to prevent large fluctuations in exchange rates [23].

In order to compare prices among the eight countries during the five years study period, sales volumes of the study drugs for each country were obtained from the IQVIA global sales data from 2013 to 2017. The sales volume in the listing year was replaced with the sales volume in the following year in order to adjust for the lower sales volume in the year of listing in the drug formulary.

### 2.3. Price Comparison According to Price Indices

The prices of the study drugs were compared among A7 countries: USA, UK, Germany, France, Italy, Switzerland, and Japan. Three volume-weighted price indices: Laspeyres, Paasche, and Fisher index and the unweighted price index were calculated using Korea as the base country [15,24,25].

The Laspeyres price index was calculated using the base country’s quantity as the weight:

$$\mathrm{Laspeyres}\;\mathrm{price}\;\mathrm{index}\;({\mathrm{PI}}_{L})=\frac{\sum_{j=1}^{n}\sum_{i=1}^{n}\begin{array}{c} \left(\mathrm{Price}\;\mathrm{of}\;\mathrm{drug}\;i\;\mathrm{in}\;\mathrm{country}\;j\right)\times \left(\mathrm{Quantity}\;\mathrm{of}\;\mathrm{drug}\;i\;\mathrm{in}\;\mathrm{Korea}\right) \end{array}}{\sum_{i=1}^{n}\begin{array}{c} \left(\mathrm{Price}\;\mathrm{of}\;\mathrm{drug}\;i\;\mathrm{in}\;\mathrm{Korea}\right)\times (\mathrm{Quantity}\;\mathrm{of}\;\mathrm{drug}\;i\;\mathrm{in}\;\mathrm{Korea} \end{array})}$$

where *i* = *i*-th study drug, *j* = *j*-th study country

The Paasche index, which was weighted by the quantity sold in the comparator country, was calculated as:

$$\mathrm{Paasche}\;\mathrm{price}\;\mathrm{index}\;({\mathrm{PI}}_{P})=\frac{\sum_{j=1}^{n}\sum_{i=1}^{n}\begin{array}{c} \left(\mathrm{Price}\;\mathrm{of}\;\mathrm{drug}\;i\;\mathrm{in}\;\mathrm{country}\;j\right)\times \left(\mathrm{Quantity}\;\mathrm{of}\;\mathrm{drug}\;i\;\mathrm{in}\;\mathrm{country}\;j\right) \end{array}}{\sum_{i=1}^{n}\begin{array}{c} \left(\mathrm{Price}\;\mathrm{of}\;\mathrm{drug}\;i\;\mathrm{in}\;\mathrm{Korea}\right)\times (\mathrm{Quantity}\;\mathrm{of}\;\mathrm{drug}\;i\;\mathrm{in}\;\mathrm{country}\;j \end{array})}$$

The Fisher index was measured by calculating the geometric mean of the Laspeyres and Paasche price indices:

$$\mathrm{Fisher}\;\mathrm{price}\;\mathrm{index}\;({\mathrm{PI}}_{F})=\sqrt{\begin{array}{c} {\mathrm{PI}}_{L}\times {\mathrm{PI}}_{P} \end{array}}$$

The unweighted price index was calculated as the ratio of drug prices in the comparator country to those in Korea without consideration of the quantity sold in the comparator country:

$$\mathrm{Unweighted}\;\mathrm{price}\;\mathrm{index}\;({\mathrm{PI}}_{U})=\frac{\sum_{j=1}^{n}\sum_{i=1}^{n}\mathrm{Price}\;\mathrm{of}\;\mathrm{drug}\;i\;\mathrm{in}\;\mathrm{country}\;j}{\sum_{j=1}^{n}\sum_{i=1}^{n}\mathrm{Price}\;\mathrm{of}\;\mathrm{drug}\;i\;\mathrm{in}\;\mathrm{Korea}}$$

Price indices were calculated using the following prices: (a) drug price in each study country, (b) average price among A7 countries, and (c) lowest price among A7 countries.

### 2.4. Budget Impact Analysis

**Figure 2:**
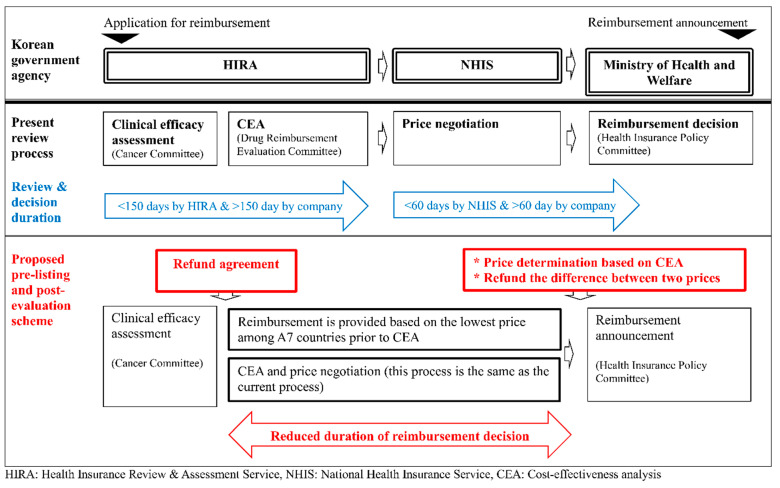
Comparison between the present review process and the proposed pre-listing and post-evaluation process.

The current reimbursement system, which determines the price of a drug based on the results of a cost effectiveness analysis, takes significantly longer from approval to reimbursement compared to that of foreign countries. This study proposes the so-called pre-listing and post-evaluation scheme, which initially reimburses a drug at the lowest price used among A7 countries. After this initial step, the actual price is determined based on the results of cost effectiveness analysis which is performed with reduced uncertainty and more real-world evidence. A pharmaceutical company pays back the difference between the temporary price at pre-listing and the final price. The comparisons of the proposed scheme to the current review process is illustrated in Figure 2.

The budget impact of implementing the proposed pre-listing and post-evaluation scheme compared with the current reimbursement process was calculated under the assumption that this proposed scheme could shorten the reimbursement decision process by one year. The annual budget impact of the pre-listing and post-evaluation scheme was calculated as the price difference between the initial list price of each study drug in Korea and their international reference prices such as the average price or the lowest price among A7 country at the time of licensing in Korea multiplied by the quantities sold obtained from IQVIA data. The price at the time of a drug’s approval in Korea was adjusted for budget impact analyses using the consumer price index of each country. Therefore, the budget impact was the sum of the price difference between the list price in Korea and the lowest price of each study drug among the study countries. The annual sales value in Korea for one year after listing was estimated using IQVIA data from 2013 to 2017. 

## 3. Results

The average time between approval and listing (reimbursement) in Korea was 22.59 months with a standard deviation of 17.08 months. List prices in different countries are presented in Table 2. USA prices were the highest for all 34 drugs, and Korean prices were the lowest with a few exceptions. Table 3 shows a comparison of four price indices between Korea and A7 countries based on the currency exchange rate and PPP. When the Laspeyres index was applied using the currency exchange rate, drug prices were higher by 2.78 (USA), 2.20 (Germany), 2.17 (Italy), 1.47 (Japan), 1.45 (Switzerland), 1.29 (France), and 1.17 (UK) compared with the prices in Korea (1.00). Although the price index varied depending on the type of price index, no large changes in drug prices were observed between countries. When the PPP was used, prices in Korea were lower than those in the USA, Germany, Italy, and Japan (range: 1.92–2.13) but higher than those in the UK, France, and Switzerland (range: 0.72–0.99).

**Figure 3:**
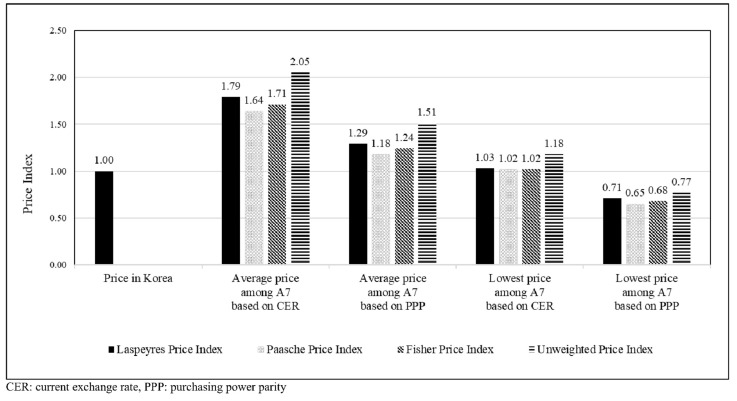
Price indices of anticancer drugs based on the currency exchange rate and purchasing power parity of the study countries (A7).

Figure 3 shows that the average prices among A7 countries were higher than the prices in Korea based on the currency exchange rate (range: 1.65–1.79) or PPP (range: 1.18–1.24). The prices in Korea were almost similar to the lowest prices among A7 countries based on the currency exchange rate (range: 1.02–1.03) but were higher than the lowest prices among A7 countries based on the PPP (range: 0.65–0.71). The trends of the unweighted price index in Korea were similar to those of volume-weighted price indices.

The budget impact of the pre-listing and post-evaluation scheme on payers is shown in Table 4. The sales values of all anticancer drugs in Korea as well as the 34 selected study anticancer drugs increased gradually from 2013 to 2017, reaching 1192 million USA dollars and 257 million USA dollars in 2017, respectively. When the lowest price among the A7 countries was applied as the reference price because this price was closest to the Korea’s drug price, the difference in sales values between Korea’s drug price and the reference price was 8.14 million USA dollars, which was 0.68% of the total estimated anticancer drug sales in Korea for 2017. On the other hand, a higher cost at 10.71% of the total anticancer drug sales for 2017 was obtained when applying the average price among A7 countries, which differed the most from Korea’s drug price.

## 4. Discussion

When the currency exchange rate was applied, anticancer drug prices were lower in Korea than in the A7 countries (price index range: 1.05–2.78) depending on the country and type of price index. The prices in Korea were similar (range: 1.02–1.03) to the lowest prices among the A7 countries. When the PPPs were applied, the prices in Korea were lower than those in the USA, Germany, Italy, and Japan (index range: 1.09–2.13), but higher than those in the UK, France, and Switzerland (index range: 0.72–0.99). When the lowest prices among A7 countries were used, the prices in Korea were higher than those in the A7 countries (index range: 0.65–0.71). This trend may be reflected by the situation in Korea, including fluctuation of the currency exchange rate and the relatively high cost of living. Thus, using currency exchange rates to make international price comparison is suggested because exchange rates determine the innovator firm’s actual net revenue from foreign sales [26]. In addition, the Korean government made reimbursement decisions of new drugs based on the prices of A7, which caused a downward convergence on the prices of new drugs. With the increasing use of external reference pricing, a low price for a new product on a given market could affect the pricing strategy of a company elsewhere [27]. A recent study found that among 29 European countries adopting an external reference pricing system, 15 countries used the average price, and seven countries used the lowest price [28]. European prices decrease over time, which require regular revisions [27]. The prices of anticancer drugs in Korea were lower than the average prices among A7 countries.

The weighted price indices differed considerably from unweighted price indices, and the findings are consistent with those of previous studies [15,17,29]. As unweighted indices are strongly influenced by extreme prices, weighted indices are considered more reliable and robust [30,31]. The lower weighted price indices compared with unweighted ones in this study may be attributed to the reduced impact of highly expensive drugs with small quantities sold. In comparison with the Laspeyres price index of anticancer drugs in Korea, the index in the USA was higher (2.78), followed by that in Germany (2.20), Italy (2.17), and Japan (1.47). Price differences between other countries and the USA, where most anticancer drugs were launched first, are consistent with the findings of previous studies [25,32,33]. The results showing the second highest price index in Germany and lowest price index in the UK are also in agreement with the findings of a recent study comparing the ex-factory prices of 31 anticancer drugs between European countries [34]. A study comparing the drug prices in 15 countries found that the overall drug prices in Korea were the third highest. However, when restricted to anticancer drugs and immunomodulators, both the unweighted and Laspeyres indices were lower in Korea than in the comparator countries, which are consistent with our results [24].

Price comparison based on the PPP is important when comparing the prices of goods across countries with consideration of variations in national income levels and purchasing power [35]. In previous studies, using the PPP instead of the normal exchange rate tended to increase drug prices in low- and middle-income countries and decrease drug prices in high-income countries [24]. In this study, the price indices in foreign countries were significantly decreased when the PPP was used, which may be attributed to the high economic level of the countries being studied. In a study that compared the drug prices of the top-rated components of claims among 15 countries based on the PPP, the unweighted price index was similar or slightly lower in Korea than in the comparator countries; however, the volume-weighted Laspeyres price index was higher. In a study that compared the prices of Alzheimer’s drugs based on the PPP with those based on the normal exchange rate, prices were decreased by 28%, 20%, and 18% in Switzerland, France, and the UK, respectively, but increased by 30% in Korea [36]; this was similar to our findings showing the higher relative prices in Korea.

The prices of new drugs in Korea are generally lower than those in OECD countries due to complex factors including a positive list system, a lower incremental cost-effectiveness ratio threshold, and stringent government-led price negotiations. Since the introduction of the positive list system, which requires cost-effectiveness analysis, the prices of new drugs have been much lower in Korea than in OECD countries [37]. The proportion of reimbursement of new anticancer drugs licensed in Korea is less than half of the OECD’s reimbursement average of 62%. In order to accelerate access to innovative drugs, the Korean government has implemented policies such as a risk-sharing agreement, a cost-effectiveness analysis waiver track, and drug-price negotiation exemption. However, these policies were only applied to a few drugs because of the narrow eligibility criteria.

Managed entry agreements have been implemented in several countries; however, there are still disagreements regarding taxonomies and terminologies, and features and names are different according to the country [9,38,39]. In comparison with performance-based agreements, financial-based agreements including refunds, rebates, price-volume agreements, and dose capping are used in at least two-thirds of OECD countries and European Union (EU) member states because of their simplicity [9,40]. Performance-based agreements, in which the final reimbursement is dependent on future evidence of effectiveness or budget impact, are used in more than half (56%) of OECD countries and EU member states [9,10,41]. The Korean government implemented a risk-sharing agreement, which is only applicable to anticancer drugs and orphan drugs that lack alternatives, to improve patient access to new drugs since December 2013. However, only 33 drugs have been listed under the risk-sharing agreement; 32 drugs were contracted with finance-based agreements such as refunds or expenditure caps, and one drug was contracted with a performance-based agreement [42].

The lengthy reimbursement review process is one of the biggest barriers to the accessibility of new anticancer drugs. The anticancer drugs examined in this study were reimbursed at a median time of 669 days (mean = 687 days) after market approval. Although there are large variations in the time to reimbursement compared with that in other countries, the median period is significantly longer in Korea than in other countries (ranging from 89 days to 422 days) [13,14]. Overall, variations in the time of drug approval to patient access may be attributed to patient preferences, assessment criteria, and reimbursement schemes. Implementing a new review process that can shorten the reimbursement decision time and expedite patient access to new drugs in South Korea is crucial for patient survival and alleviating societal economic burden.

The present study proposes a new scheme called the pre-listing and post-evaluation scheme for new anticancer drugs in Korea to improve patient accessibility. Under this scheme, new drugs can be reimbursed at the lowest price among A7 countries, which is the closest approximation to the price of anticancer drugs in Korea as demonstrated by our results. Furthermore, the lowest price among A7 countries has already been used in Korea for the external reference pricing of some oncology drugs with few alternatives, which are exempted from cost-effectiveness analysis [11]. Afterwards, the actual price is determined based on cost effectiveness analysis of the drugs using accumulated real-world evidence.

The pre-listing and post-evaluation scheme, which is a type of performance-based managed entry agreement or risk-sharing agreement, can play a vital role in improving the accessibility of new drugs. Pharmaceutical companies and the government share the financial burdens and uncertainties regarding clinical outcomes. Anticancer drug expenditures under this scheme were calculated at 0.68% of the total anticancer drug sales in 2017. An increased financial burden and the potential discontinuation of the supply of medications would not be major obstacles to the implementation of this proposed scheme. At the beginning of negotiations, the Korean government and pharmaceutical companies can sign a contract after discussing and agreeing on aspects such as the indication and coverage of the medication, the maximum cap for sales volume, sources of data that can be used to evaluate performance, the transparency of the review process, conditions for rebate amounts, and conditions for continuing supply of a drug for a certain period if a company withdraws a drug from the drug formulary. Listing new drugs under the public health insurance system without economic evaluation may raise concerns about the efficient allocation of resources and agreement of pricing; however, the non-reimbursement of new anticancer drugs can be a significant barrier to patient accessibility [43].

In several OECD countries, the use of a special fund is another method of increasing patient access to innovative drugs [44,45]. In the UK, The Cancer Drugs Fund provides £200 million per year for cancer treatments that were not appraised by the National Institute for Health and Care Excellence (NICE) [46]. The Australian Government provides fully subsidized access to essential medicines through the Life Saving Drugs Program designed for eligible patients with rare and life-threatening diseases [47]. Italy has allocated €80 million to an innovative medicine fund [48]. Although these funds function by providing immediate access and short-term gains for patients and companies, it is possible that a fund specifically created for anticancer drugs may act as a disincentive for manufacturers to offer discounts to the National Health Service through a patient access scheme in the UK [49].

Although this study is the first study to compare the prices of anticancer drugs based on various prices and quantities sold in real-world settings, some limitations of the present study should be pointed out. The present study adjusted ex-factory prices after considering the allowed margin and confidential discount levels of each country, which were surveyed by the HIRA. However, the calculated prices in the present study may differ from the true ex-factory prices because it is impossible to determine the actual confidential discounts and hidden rebates. Although drug prices are influenced by the launch date, patent expiration, and the type of price regulation, the present study could not take into account all of these factors [30]. Finally, the sales volume of medications was obtained from IQVIA data; however, IQVIA data may not cover the total sales volume in A7 countries.

Despite the limitations described above, this study has methodological strengths such as the estimation of the ex-factory price, which is free of distribution margins/rebates and differences between the wholesale price and retail price. This study also compared three different volume-weighted price indices, which were calculated using the price in each country and the average price and lowest price among the A7 countries. This study provided specific evidence supporting the feasibility of a pre-listing and post-evaluation reimbursement review process to facilitate access to novel anticancer medicines. Our findings could help policymakers develop diverse and flexible reimbursement strategies to improve patient access to novel anticancer drugs without compromising affordability. Given the trends in the global pharmaceutical industry, more high-priced anticancer drugs targeting a particular patient group could be developed in the future. The large increase in the elderly population will increase the demand for these drugs. The pre-listing and post-evaluation scheme for new anticancer drugs, which is a performance-based managed entry agreement, could be regarded as a way of preparing for dynamic situations in the future. It could allow payers to manage insurance finances efficiently according to the principles of pricing and reimbursement prescribed by the system and allow patients access to new anticancer drugs quickly. 

## 5. Conclusions

The prices of anticancer drugs in Korea were lower than those in the A7 countries and were similar to the lowest prices among the A7 countries; however, there were small variations in the prices depending on the types of price indices and currency conversion methods. This study introduces a pre-listing and post-evaluation scheme in which a drug is reimbursed based on the lowest price among the A7 countries. This proposed scheme, which is a type of performance-based managed entry agreement, can expedite patient access to novel anticancer drugs with a reduced burden of budget on the government.

## Figures and Tables

**Table 1:** Characteristics of the selected study cancer drugs.

Generic Name (Brand Name)	Initial Indication	Manufacturer	Strength	Approval Date in Korea	Listing Date for Reimbursement	Price at Listing date (US$)	Duration for Listing * (Months)
Dasatinib (Sprycel^®^)	Leukemia	Bristol-Myers Squibb	20 mg	25/1/2007	6/2008	20.60	16
			50 mg	25/1/2007	6/2008	39.50	16
			70 mg	25/1/2007	6/2008	47.22	16
			80 mg	27/10/2016	2/2017	47.97	4
			100 mg	14/4/2011	10/2011	57.14	6
Lapatinib (Tykerb^®^)	Metastatic breast cancer	Novartis	250 mg	30/7/2007	3/2010	11.65	31
Bevacizumab (Avastin^®^)	Metastatic colorectal cancer	Roche	100 mg/4 mL	12/9/2007	3/2014	340.95	77
			400 mg/16 mL	12/9/2007	3/2014	1108.10	77
Nilotinib (Tasigna^®^)	Chronic myeloid leukemia	Novartis	150 mg	24/12/2010	7/2012	16.92	18
			200 mg	26/10/2007	12/2011	19.79	49
Sorafenib (Nexavar^®^)	Advanced renal cell carcinoma	Bayer	200 mg	7/1/2008	5/2008	21.88	4
Fludarabine (Fludara^®^)	Chronic lymphocytic leukemia	Sanofi	10 mg tablet	4/2/2008	4/2010	24.79	26
			50 mg vial	4/2/2008	4/2010	203.14	26
Paclitaxel (Abraxane^®^)	Metastatic breast cancer	Celgene	100 mg	31/3/2008	8/2009	261.01	16
Exemestane (Aromasin^®^)	Metastatic breast cancer	Pfizer	25 mg	24/7/2008	6/2009	4.24	10
Temsirolimus (Torisel^®^)	Advanced renal cell carcinoma	Pfizer	30 mg/1.2 mL	24/10/2008	6/2011	680.87	32
Everolimus (Afinitor^®^)	Metastatic breast cancer	Novartis	2.5 mg	10/6/2011	2/2012	26.90	8
			5 mg	26/6/2009	8/2011	53.81	25
			10 mg	26/6/2009	8/2011	80.70	25
Decitabine (Dacogen^®^)	Myelodysplastic syndrome	Janssen	50 mg	27/6/2007	8/2008	663.02	13
Pazopanib (Votrient^®^)	Advanced renal cell carcinoma	Novartis	200 mg	11/8/2010	5/2011	24.47	9
			400 mg	11/8/2010	5/2011	38.64	9
Eribulin (Halaven^®^)	Metastatic breast cancer	Eisai	1 mg/2 mL	17/8/2012	6/2014	159.70	22
Ruxolitinib (Jakavi^®^)	Myelofibrosis	Novartis	5 mg	21/1/2013	5/2015	24.08	25
			15 mg	21/1/2013	5/2015	48.17	25
			20 mg	21/1/2013	5/2015	48.17	25
Degarelix (Firmagon^®^)	Advanced prostate cancer	Ferring	80 mg	18/4/2013	11/2015	117.10	30
			120 mg	18/4/2013	11/2015	146.38	30
Afatinib (Giotrif^®^)	Metastatic non-small cell lung cancer	Boehringer Ingelheim	20 mg	29/1/2014	10/2014	28.49	8
			30 mg	29/1/2014	10/2014	35.61	8
			40 mg	29/1/2014	10/2014	41.55	8
Obinutuzumab (Gazyva^®^)	Chronic lymphocytic leukemia	Roche	1000 mg/40 mL	22/9/2014	4/2017	3586.86	30
Lenvatinib (Lenvima^®^)	Thyroid cancer	Eisai	4 mg	7/10/2015	8/2017	27.39	22
			10 mg	7/10/2015	8/2017	27.39	22

* Duration for listing: elapsed time in months from the approval date by the Korean Ministry of Food and Drug Safety until the listing date for reimbursement by the Ministry of Health and Welfare.

**Table 2:** List prices of the study drugs in the study countries.

Generic Name (Brand Name)	Strength	S. Korea	U.S.	U.K.	Germany	France	Italy	Switzerland	Japan
Dasatinib (Sprycel^®^)	20 mg	18.84	134.03	27.26	41.54	33.80	NA	40.42	36.69
	50 mg	38.40	268.07	54.52	83.69	65.20	122.71	85.69	86.74
	70 mg	44.83	223.39	NA	NA	65.20	NA	85.69	NA
	80 mg	47.97	483.15	109.03	167.40	NA	245.40	NA	NA
	100 mg	56.98	483.15	109.03	167.40	130.39	245.40	175.90	NA
Lapatinib (Tykerb^®^)	250 mg	11.30	54.37	15.00	21.03	17.95	28.96	23.87	15.26
Bevacizumab (Avastin^®^)	100 mg/4 mL	297.35	910.25	316.87	395.49	NA	560.60	501.47	381.99
	400 mg/16 mL	966.43	3034.16	1207.09	1435.33	NA	2242.40	1792.44	1454.67
Nilotinib (Tasigna^®^)	150 mg	16.84	124.15	28.36	29.73	26.49	45.91	35.03	33.10
	200 mg	19.72	124.15	28.36	42.77	34.88	61.22	51.60	43.37
Sorafenib (Nexavar^®^)	200 mg	16.32	164.79	41.69	38.65	29.74	58.31	43.82	42.80
Fludarabine (Fludara^®^)	10 mg tablet	24.79	NA	26.34	NA	32.05	35.34	NA	34.25
	50 mg vial	162.50	350	195.77	91.66	NA	282.10	211.51	313.48
Paclitaxel (Abraxane^®^)	100 mg	250.79	1508.68	321.23	346.61	NA	405.40	423.60	449.40
Exemestane (Aromasin^®^)	25 mg	4.02	40.26	3.86	5.48	2.43	2.65	4.97	3.96
Temsirolimus (Torisel^®^)	30 mg/1.2 mL	626.77	2061.66	809.60	970.88	NA	1807.84	1300.29	1251.21
Everolimus (Afinitor^®^)	2.5 mg	24.21	506.06	52.23	48.10	52.18	NA	NA	64.00
	5 mg	47.17	529.33	97.93	97.43	100.59	148.92	105.15	123.99
	10 mg	69.95	529.33	116.34	140.52	129.61	211.80	139.33	NA
Decitabine (Dacogen^®^)	50 mg	578.59	2155.86	1267.76	1695.75	NA	2035.31	1605.88	NA
Pazopanib (Votrient^®^)	200 mg	23.33	110.19	24.39	33.82	27.75	50.11	36.95	37.91
	400 mg	35.92	NA	48.80	67.86	52.10	100.20	69.35	NA
Eribulin (Halaven^®^)	1 mg/2 mL	154.38	1279.2	471.40	438.24	NA	661.88	520.22	603.14
Ruxolitinib (Jakavi^®^)	5 mg	23.27	NA	33.29	44.89	38.84	65.15	39.41	33.93
	15 mg	46.53	NA	66.60	90.43	75.25	130.29	75.48	NA
	20 mg	46.53	NA	66.60	90.43	75.25	130.29	75.48	NA
Degarelix (Firmagon^®^)	80 mg	117.10	584.14	168.93	152.81	162.47	236.52	233.95	NA
	120 mg	146.38	914.51	169.76	138.41	145.28	224.48	231.42	NA
Afatinib (Giotrif^®^)	20 mg	26.97	325.22	94.36	101.45	74.21	133.87	108.17	53.45
	30 mg	33.71	325.22	94.36	101.45	74.21	133.87	108.17	78.23
	40 mg	39.33	325.22	94.36	101.45	74.21	133.87	108.17	102.49
Obinutuzumab (Gazyva^®^)	1000 mg/40 mL	3,586.86	7,160.71	4,324.84	4,373.64	NA	6,569.26	3,566.49	NA
Lenvatinib (Lenvima^®^)	4 mg	27.39	290.5	62.58	78.38	63.67	103.40	80.35	36.21
	10 mg	27.39	309	62.58	78.38	63.65	103.52	80.35	85.61

The currency rates equivalent to 1 US$ as of January 2020 were as follows: Korea (=1164.45), United Kingdom (UK) (=0.765), Germany (=0.900), France (=0.900), Italy (=0.900), Switzerland (=0.970), and Japan (=109.26).

**Table 3:** Comparison of the price indices of anticancer drugs. between Korea and the study countries.

Based on	Price Index in Each Country							
	S. Korea	U.S.	U.K.	Germany	France	Italy	Switzerland	Japan
**Currency Exchange Rate**								
Laspeyres PI	1.00	2.78	1.17	2.20	1.29	2.17	1.45	1.47
Paasche PI	1.00	2.47	1.05	1.90	1.43	1.99	1.33	1.49
Fisher PI	1.00	2.62	1.11	2.04	1.36	2.07	1.39	1.48
Unweighted PI	1.00	3.88	1.24	2.36	1.39	2.18	1.51	1.98
**Purchasing Power Parity**								
Laspeyres PI	1.00	2.13	0.78	1.59	0.99	1.64	0.80	1.11
Paasche PI	1.00	1.92	0.72	1.47	0.95	1.58	0.74	1.09
Fisher PI	1.00	2.02	0.75	1.53	0.97	1.61	0.77	1.10
Unweighted PI	1.00	2.93	0.85	1.67	0.98	1.69	0.82	1.31

U.S.: United States, U.K.: United Kingdom, PI: price index.

**Table 4:** Annual budget impact of the pre-listing and post-evaluation scheme in South Korea.

	2013	2014	2015	2016	2017	Average Annual Sales for 2013–2017
Actual total sales of all cancer drugs ^1^ ($: thousand)	$792,270	$844,509	$973,498	$1,093,892	$1,192,540	$979,342
Actual sales of 34 study drugs ($: thousand)	$84,572	$110,190	$162,546	$224,399	$257,275	$167,424
**Budget Impact for One Year**						
**Based on the Average Price Among A7**						
Sales value in 2017 ($127,687,000)						
% of total cancer drug sales	16.12%	15.12%	13.12%	11.67%	10.71%	13.04%
**Based on the Lowest Price Among A7**						
Sales value in 2017 ($8,134,000)						
% of total cancer drug sales	1.03%	0.96%	0.84%	0.74%	0.68%	0.83%

^1^ Total sales of all cancer drugs were based on drugs classified with the code number 421 or 429 by the Korean Ministry of Food and Drug Safety.

## Data Availability

The datasets used and analyzed in the study are available from the corresponding author on reasonable request.
